# Soil and seed both influence bacterial diversity in the microbiome of the *Cannabis sativa* seedling endosphere

**DOI:** 10.3389/fpls.2024.1326294

**Published:** 2024-02-21

**Authors:** Christopher R. Dumigan, Michael K. Deyholos

**Affiliations:** Department of Biology, Faculty of Science, University of British Columbia, Kelowna, BC, Canada

**Keywords:** microbiota, hemp, endophyte, metagenome, phytobiome

## Abstract

**Introduction:**

Phytobiomes have a significant impact on plant health. The microbiome of *Cannabis sativa* is particularly interesting both because of renewed interest in this crop and because it is commercially propagated in two different ways (i.e. clonally and by seed). Angiosperms obtain a founding population of seed-borne endophytes from their seed-bearing parent. This study examines the influence of both seed and soil-derived bacteria on the endospheres of cannabis seedlings of both hemp- and drug-types.

**Methods:**

A multi-factorial metagenomic study was conducted with three cannabis genotypes and two soil sources, which were tested both before and after autoclave sterilization. Seedlings were grown on soil, then rinsed and surface-sterilized, and 16S rDNA amplicons from seedling endophytes were sequenced, taxonomically classified, and used to estimate alpha- and beta-diversity in Qiime2. The statistical significance of differences in seedling microbiomes across treatments was tested, and PiCRUST2 was used to infer the functional relevance of these differences.

**Results:**

Soil was found to have a profound effect on the alpha-diversity, beta-diversity, relative abundance, and functional genes of endophytic bacteria in germinating cannabis seedlings. Additionally, there was a significant effect of cannabis genotype on beta diversity, especially when genotypes were grown in sterilized soil. *Gammaproteobacteria* and *Bacilli* were the two most abundant taxa and were found in all genotypes and soil types, including sterilized soil.

**Discussion:**

The results indicated that a component of cannabis seedling endosphere microbiomes is seed-derived and conserved across the environments tested. Functional prediction of seedling endophytes using piCRUST suggested a number of important functions of seed-borne endophytes in cannabis including nutrient and amino acid cycling, hormone regulation, and as precursors to antibiotics. This study suggested both seed and soil play a critical role in shaping the microbiome of germinating cannabis seedlings.

## 1 Introduction

Endophytes are microorganisms that live inside plant tissues. Due to their intimate associations with their hosts, they can have powerful effects on plant physiology (Hardoim et al., 2008; Johnston-Monje and Raizada, 2011; Hardoim et al., 2012, Hardoim et al., 2015; Truyens et al., 2015). Endophytes can promote plant growth by providing nutrients, increasing nutrient uptake, modulating and secreting phytohormones, and defending against pathogens of plants (Hu et al., 2003; Johnston-Monje and Raizada, 2011; Mousa et al., 2016; Shehata et al., 2017). Plants appear to select specific endophytes, especially during seedling emergence, and these endophytes may be vectored by seeds across generations to protect seedlings against environmental stresses (Truyens et al., 2015; Pitzschke, 2016; Shahzad et al., 2018; Abdelfattah et al., 2021; Rochefort et al., 2021). For example, significant portions of endophyte species in juvenile maize plants are seed-derived and inherited from their seed-bearing parent (Johnston-Monje et al., 2014, Johnston-Monje et al., 2016).

Plant-associated microbiota can be derived from both the environment and from the parent, although the relative contribution of each is not always clear (Aleklett and Hart, 2013). Some microbial endophytes appear to be widely conserved in angiosperms, independent of soil environments, and even when grown on sterile substrates. This suggests that at least some plant-associated microbes are seed-derived (Johnston-Monje et al., 2014, Johnston-Monje et al., 2021). Moreover, some plants appear to have “core” microbiota that are common to most individuals of a species (Sánchez-López et al., 2018) (Johnston-Monje et al., 2014; Truyens et al., 2015; Walitang et al., 2018).

This phenomenon of seed-borne microbial inheritance in cannabis was recently demonstrated for the first time (Dumigan and Deyholos, 2022). This study showed that hemp and drug-type cannabis cultivars grown to seed at several locations in western Canada vectored bioactive and antifungal endophytic bacteria to the next generation of seedlings. Furthermore, specific bacteria from the class Bacilli were found as endophytes of all cannabis genotypes examined. However, this previous study was limited to culturable microorganisms, and was conducted in axenic conditions, so the effect of soil on the endophytic microbiome was not tested. Drug-type cannabis plants intended for medical and recreational markets in Canada are typically grown in soilless medium. This provides growers more control over pathogens that can be transferred from soil. However, it also restricts potentially beneficial microorganisms that can be soil derived and may inadvertently alter the microbiome of cannabis plants is structured. An important question arises from this largely unstudied topic: what are the relative effects of soil and seed-derived factors on the composition of the seedling endophyte community in cannabis?

In the current study, we hypothesized that soil will have a significant impact on the microbiome of cannabis seedling endosphere, while a component of the cannabis seedling endosphere bacteria will be derived from seed-borne bacteria and independent of soil conditions. We tested this hypothesis using 16S-based amplicon metagenomics to compare the effects of two soil types, with or without sterilization, on the composition of the endosphere microbiome in three different cannabis genotypes.

## 2 Materials and methods

### 2.1 Sources of seeds

*Cannabis sativa* drug-type “BC Big Bud” seeds were purchased from a commercial supplier in Qualicum Beach, BC, Canada. Seeds of the hemp genotypes “Katani” and “X59” were obtained from InnoTech Alberta, Vegreville, AB. All research was conducted in a Health Canada licensed facility (LIC-0IW2D2L5JY-2019). The three cannabis genotypes were selected arbitrarily, but with the intention to assay multiple different genotypes representing both hemp and drug-type crops.

### 2.2 Sources of soils

“Innotech” topsoil was collected from a field at InnoTech Alberta (Vegreville, AB) (53.504377, -112.088324) in which the hemp cultivar Silesia was been grown. “Okanagan” soil was harvested from Westpoint Vineyard in Kelowna, BC (49.821298, -119.470140) growing Chardonnay’ (*Vitis vinifera* L.) wine grape Clone 99 (121) on S04 (V*. vinifera*) rootstock. These soils were selected to represent both a soil in which cannabis has already been grown, and soil in which cannabis has not been previously grown. To sterilize the soils, a small volume of each soil type was sealed in a 2 L glass mason jar and sterilized by two successive cycles of autoclave sterilization for 55 minutes at 121°C, and the start of each cycle of autoclaving was separated by 24 hours (Carter et al., 2007). Absence of culturable microorganisms in autoclaved soil was confirmed by spreading approximately 1 g of soil from each jar on Petri dishes containing R2A agar in a laminar flow hood and inspecting plates for visible colonies after 3 days incubation at 25°C.

### 2.3 Seedling growth and experimental design

Each of four types of soil (Innotech non-sterilized, Innotech sterilized, Okanagan non-sterilized, Okanagan sterilized) were distributed into sterilized 25 mm x 200 mm borosilicate test tubes. Working in a laminar flow hood, soil was added to fill tubes to 5 cm height (approximately 25 mL of soil). One seed of each of the three genotypes (BC Big Bud, Katani, X59) was placed in the soil in each tube, watered with 1 mL sterile RO water, and the tubes were sealed with cotton balls. The tubes were placed under fluorescent light at 8250 lux, 7 days prior to removal of the seedlings. There were four replicates for each combination of the four soil types and three cannabis genotype, thus 48 seedlings per replicate. Two independent replicates were conducted for a total of 96 seedlings analyzed.

### 2.4 Seedling surface sterilization and DNA extraction

After removal from soil, seedlings were first washed with filter-sterilized 0.1% Triton-X for 10 min, and then 3% sodium hypochlorite for 20 min. Samples were drained and rinsed with autoclaved, deionized water, followed by treatment with 70% ethanol for 10 minutes. Samples were rinsed 5 times to remove ethanol before being frozen for DNA isolation. An aliquot of the final rinse was plated on R2A medium to test sterility. DNA from whole 7-day old seedlings (including both roots and shoots) was isolated using the Qiagen DNeasy Plant Pro kit according to the manufacturer’s instructions and using a Powerlyser24 homogenizer at 1500 rpm for 1 min, then 2000 rpm for 1 min.

### 2.5 16S rRNA amplicon sequencing

The V4 hypervariable region of the 16S rRNA gene was amplified using barcoded primers with Illumina adapters: 515FB: GTGYCAGCMGCCGCGGTAA, 806RB: GGACTACNVGGGTWTCTAAT. 1 μM pPNA and 1 μM mPNA (PNA Bio Inc, Newbury Park, CA) were included in the PCR to inhibit amplification of plant chloroplast and mitochondria sequences. The PCR cycle was as follows: 95°C for 5 min, followed by 35 cycles of 95°C for 30 s, 55°C for 30 s and 72°C for 50 s, and then extended at 72°C for 10 min. Amplicons were visualized on a 1% TAE agarose gel. Amplicons were cleaned using Mag-Bind^®^ TotalPure NGS magnetic beads (Omega), followed by indexing PCR with i7 and i5 primers (95°C for 3 minutes followed by 8 cycles of 95°C for 30 seconds, 55°C for 30 seconds, 72°C for 30 seconds, followed by 72°C for 5 minutes). Indexed amplicon libraries were bead-cleaned a second time before quantification using a Nanodrop spectrophotometer. Equimolar samples were then pooled and sent to Genome Quebec for DNA sequencing using the Illumina MiSeq Reagent Kit v3 (2x300 cycles).

### 2.6 Data analysis

Demultiplexed reads were analyzed using the qiime2-2022.2 pipeline running on a CentOS Linux 7 server (8 cores, 124 GB RAM, Python: 3.8.12) (Bolyen et al., 2019). Paired-end reads were trimmed and truncated to maximize sequence quality, dereplicated and denoised into amplicon sequence variants (ASVs) using DADA2 (Callahan et al., 2016). The resulting feature table was taxonomically classified using the q2-feature-classifier and the machine learning based classification method scikit-learn trained against the Green Genes Database and with primers 515F and 806R (gg-13-8-99-515-806-nb-classifier.qza) (DeSantis et al., 2006; Caporaso et al., 2011). Chloroplast, mitochondria, and taxa without phylum-level classification were removed using qiime2 filter-seq. Sequences were aligned using the MAFFT plugin and phylogenetic trees were conducted using the FastTree plugin (Katoh et al. 2002; Price et al., 2010). Alpha and beta diversity was assessed using qiime diversity core-metrics-phylogenetic plugin following sample rarefaction to an even sampling depth. Principle coordinate analyses were conducted using qiime2 with Bray-Curtis diversity metrics. PERMANOVA and PERMDISP were used to assess significance in the differences in seed-borne cannabis seedling endosphere microbiomes across treatments (Anderson et al., 2006; Anderson, 2017) (**Supplementary File S1**). ANCOM differential abundance testing was conducted using Qiime2 rarefied to minimum frequency of 50 (–p-min-frequency 50) (**Supplementary File S2**). Significance was tested for differentially abundant bacteria using qiime2 by running a series of pairwise tests in which the W statistic was a count of the number of times the null hypothesis that there is no difference was rejected. PiCRUST2 (Phylogenetic Investigation of Communities by Reconstruction of Unobserved States) was used to infer functional genes in treatments based on 16S marker sequences and the corresponding genomic data for the respective amplicon sequence variants (ASVs) (Douglas et al., 2013) (**Supplementary File S3**). Qiime2 artifacts were converted to publication quality plots using Qiime2R plugin and RStudio version 4.1.2. Metadata for the Qiime2 analysis are provided in **Supplementary File S4**.

## 3 Results

### 3.1 Effect of seed and soil on cannabis seedling microbiomes

**Figure 1:**
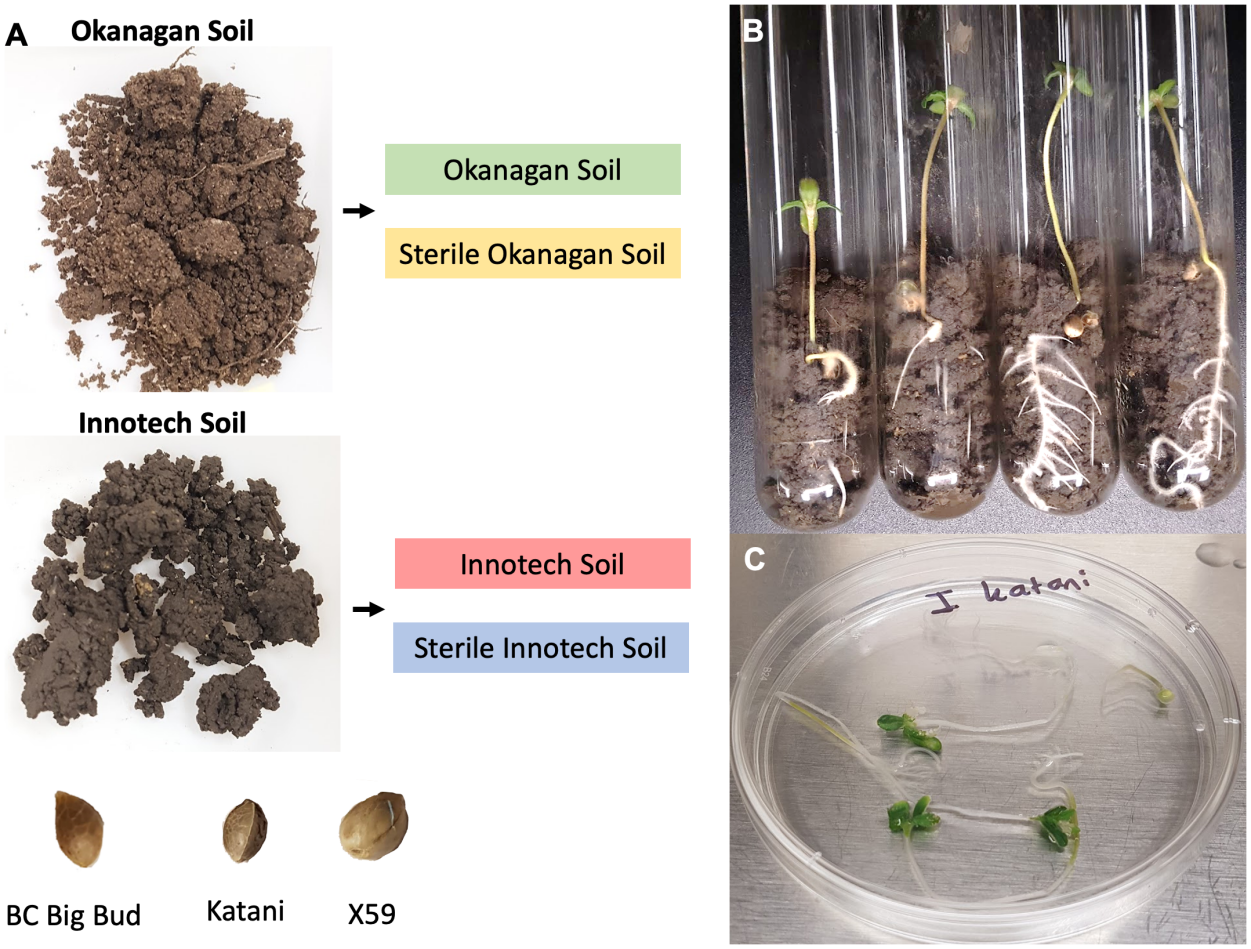
Experimental overview. **(A)** Three cannabis genotypes (BC Big Bud, Katani, X59) were each grown in four different combinations of soil type (origin: Okanagan or Innotech; treatment: sterilized or non-sterilized) in two independent trials. **(B)** Plants were grown for 7 days prior to **(C)** surface sterilization. DNA isolation, and amplicon metagenomic sequencing followed but are not shown.

To investigate the relative contributions of both seed and soil to the microbiota of the cannabis seedling endosphere, we profiled the microbiomes of seedlings from three different genotypes under four different soil conditions. The cannabis genotypes that were selected for testing were: BC Big Bud (indoor grown drug-type); X59 (field-grown hemp); and Katani (field-grown hemp). These genotypes were selected to represent diverse germplasm. Soil was collected from the surface of two diverse sources: an eluviated eutric brunisol from a vineyard in the Okanagan valley (BC, Canada), and black chernozem from a hemp field at a research station in Vegreville (AB, Canada). Non-sterilized and sterilized (autoclaved) soils from each location were used in a full multifactiorial design (n=4, for each of the twelve combinations of seed genotype x soil source x sterilization status; replicated twice for a total of 96 seedlings). (**Figure 1**).

**Figure 2:**
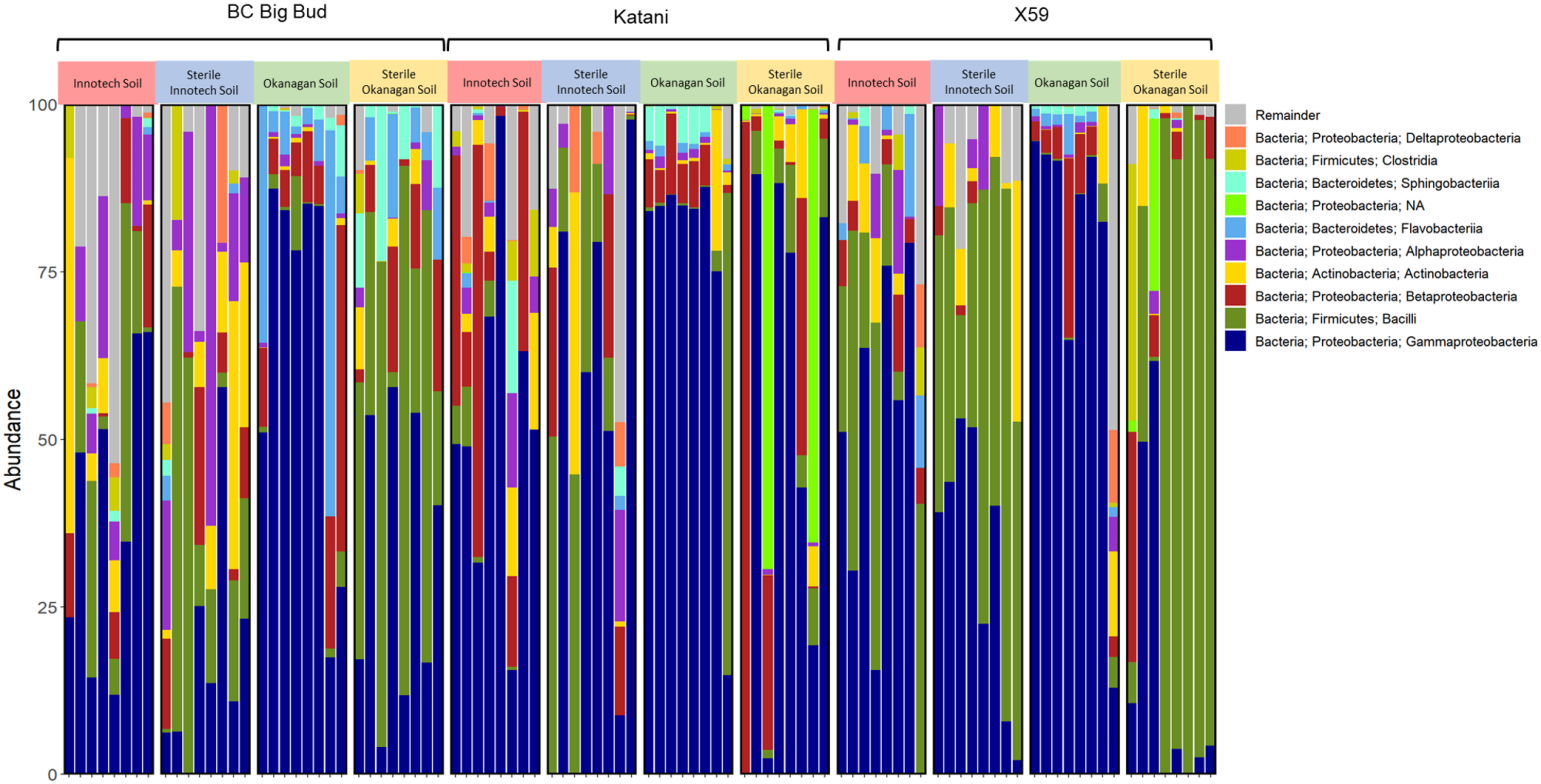
Taxonomic bar plot of cannabis seedling endosphere bacterial phyla. Amplicon sequencing was used to determine relative abundance of phylum level ASVs (amplicon sequence variants) from 7-day old cannabis seedling endospheres. Seedlings are from three different cannabis genotypes and four soil conditions.

**Figure 3:**
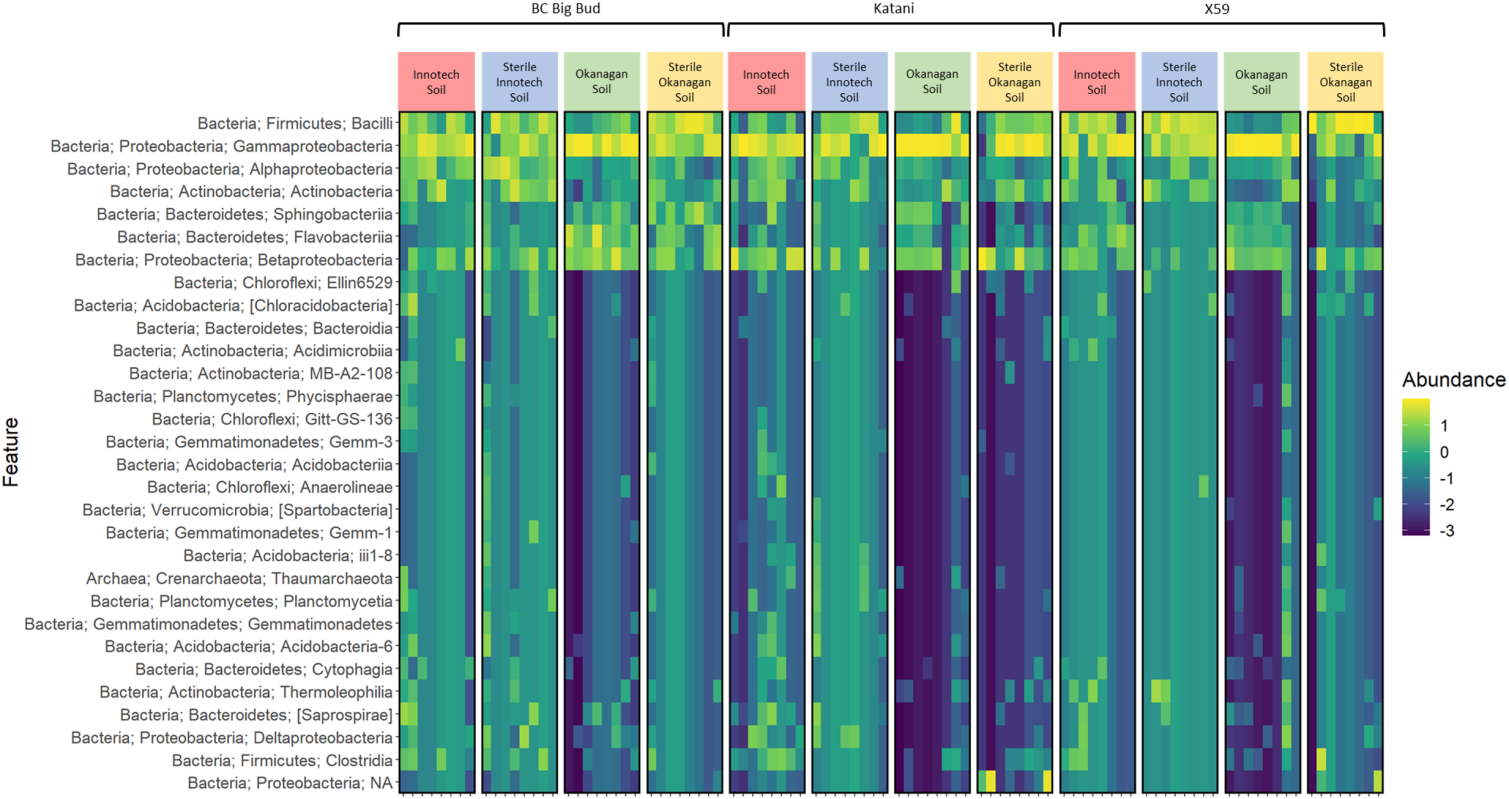
Heatmap of abundance of bacterial taxa in cannabis seedling endospheres. Amplicon sequencing was used to determine abundance of phylum level ASVs (amplicon squence variants) from 7-day old cannabis seedling endospheres. Seedlings are from three different cannabis genotypes and four soil conditions.

Amplicon sequencing of each of the 96 seedlings yielded 15.1 million reads with an average quality score of 32.1. After denoising with DADA2, classifying, and filtering, a total of 1,290 unique features remained with a total frequency of 1789601. Among all reads that were classified, *Gammaproteobacteria* and *Bacilli* were the two most abundant taxa and were found in all genotypes and soil types (**Figures 2**, **3**). Within genotypes, the abundance of *Gammaproteobacteria* and *Bacilli* appeared to be inversely correlated across soil types (**Figure 2**). Additionally, *Betaproteobacteria* were widespread, although at generally lower abundance than either *Gammaproteobacteria* or *Bacilli* (**Figure 2)**.

#### 3.1.1 Effect of soil sterilization on alpha-diversity

**Figure 4:**
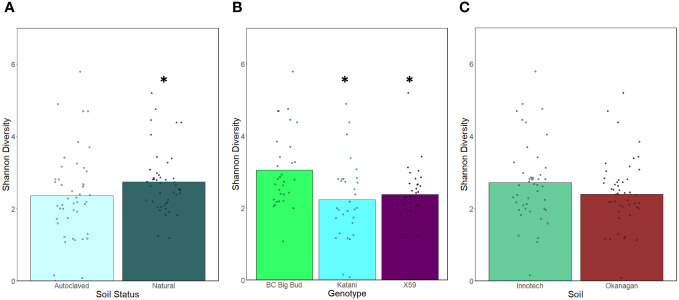
Effect of soil autoclaving, soil origin, and cannabis genotype on alpha-diversity of cannabis seedling endospheres. Shannon diversity was compared using a Kruskal-Wallis (pairwise) test. **(A)** Soil sterilization resulted in a significant drop in alpha-diversity when pooling genotypes and soils (p=0.03730). **(B)** Katani and X59 had significantly lower alpha diversity when all soil treatments were considered together (p= 0.00333, 0.01516 respectively). **(C)** Innotech and Okanagan soil showed no significant difference in alpha-diversity when all genotypes and soil sterilization treatments were pooled p=0.19651). Asterisks indicate significant effects.

Seedlings grown in non-sterilized soils had significantly higher endosphere alpha-diversity as compared to sterilized soils when all cannabis genotypes and soil sources were considered together (p=0.03730) (**Figure 4A**). This shows that the soil inoculum is a significant factor in microbial diversity in the seedling endospheres. When the effect of seedling genotype was compared across all soil conditions, BC Big Bud had significantly higher alpha-diversity compared to either Katani or X59 (p= 0.00333, 0.01516 respectively) (**Figure 4B**). There was no significant difference between Katani and X59 (p=0.341958). There was no significant difference in alpha-diversity between seedlings grown in Innotech and Okanagan soil, when all genotypes and both sterilized and non-sterilized treatments were considered together (p= 0.19651)] (**Figure 4C**).

**Figure 5:**
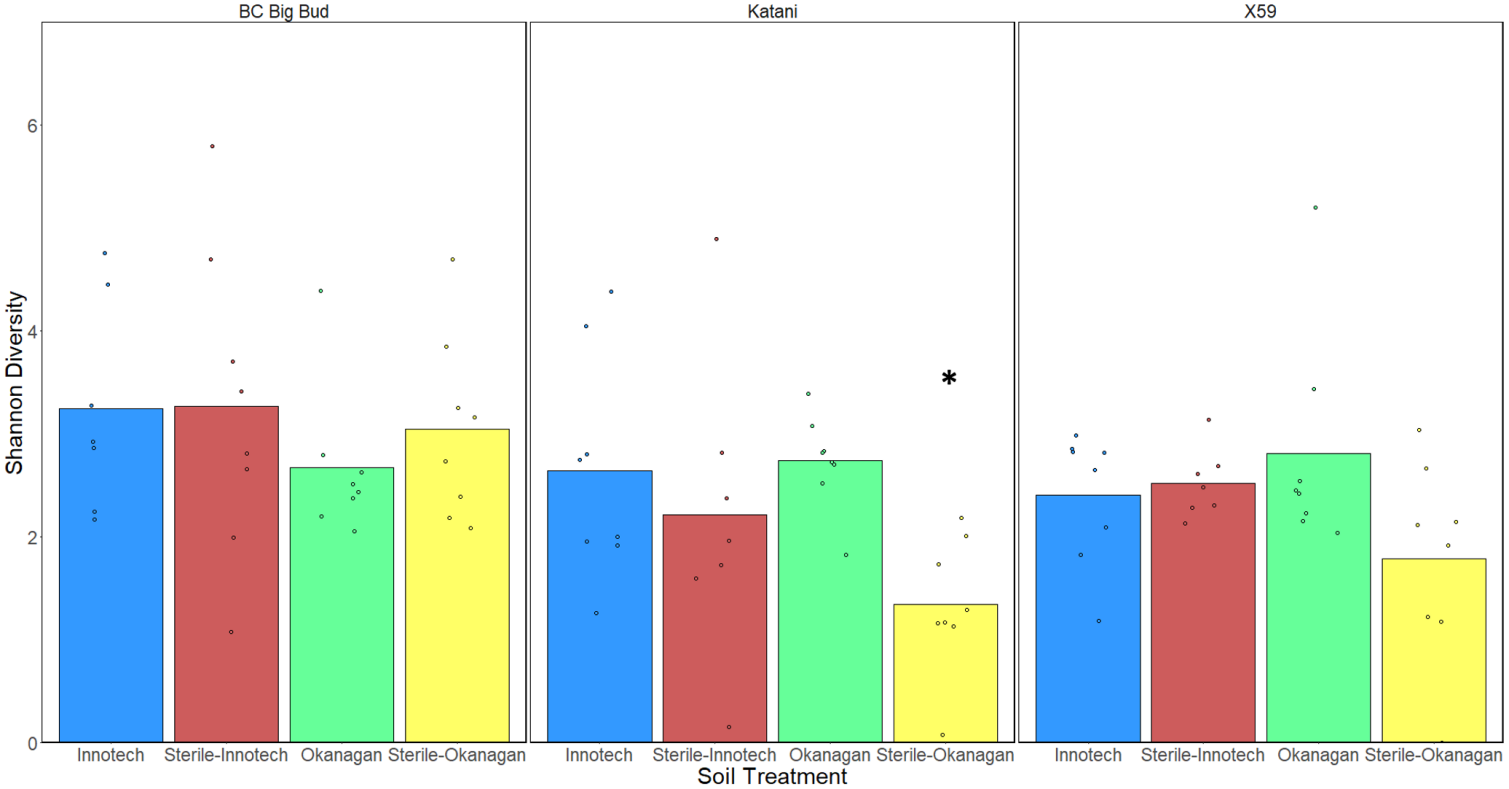
Effect of soil treatment on alpha-diversity of individual genotypes of cannabis seedling endospheres. Shannon diversity was compared using a Kruskal-Wallis (pairwise) test. Only Katani plants grown in Okanagan soil showed a reduction in alpha-diversity from soil-sterilization (p=0.001629), as indicated by the asterisk.

When the effect of soil sterilization was considered on each cannabis genotype individually, only Katani seedlings grown in Okanagan soil showed a significantly lower alpha-diversity in sterilized as compared to non-sterilized soils (p=0.001629) (**Figure 5**). Katani seedlings grown in Innotech soil did not show a significant difference effect of soil sterilization. Neither did any other genotype of cannabis tested show a significant difference in alpha-diversity across sterilized and non-sterilized soils from the same soil source.

#### 3.1.2 Effect on beta-diversity

**Figure 6:**
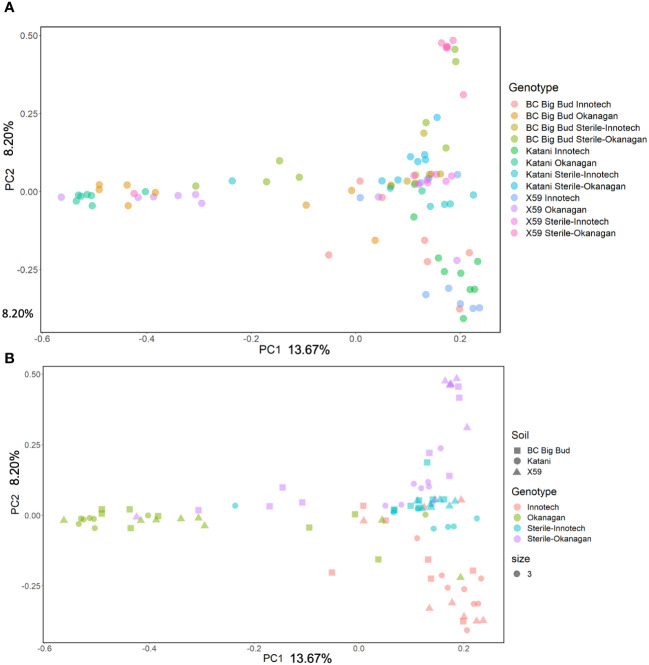
Principle Coordinate Analysis of Bray-Curtis Distances in Experiment 2. **(A)** All treatments individually coloured on PCOA. **(B)** Soil treatments coloured to show clustering of samples within soil groups.

Soil source, soil sterilization, and cannabis genotype all had a significant effect on the bacterial beta-diversity of the seedling endosphere as measured by the Bray-Curtis distance (**Figure 6**; **Supplementary Table 1**). Notably, the Bray-Curtis distance did not differ between genotypes when they were grown on non-sterilized Innotech soil (p=0.189, 0.14, 0.124). Conversely, in Okanagan soil, the Bray-Curtis distance did differ significantly between cannabis genotypes (p=0.032, 0.002, 0.001) (**Figure 6**; **Supplementary Table 2**). This indicates that in the case of Innotech soil, but not Okanagan soil, the soil-derived microorganisms strongly contributed to endosphere microbiome structure and limited the influence of seed-vectored bacteria.

Different genotypes grown in the sterilized soil from the same source had significantly different beta-diversity, showing a genotype effect on microbial diversity in cannabis seedling endospheres when soil inoculum was absent (PERMANOVA p= 0.001, PERMDISP p= 0.727) (**Figure 6**; **Supplementary Table S1**). Furthermore, soil sterilization had a significant effect on the beta-diversity of seedling endospheres across all three genotypes, demonstrating the importance of live soil bacteria in shaping juvenile cannabis endospheres. Finally, even when sterilized, soils from different sources had a significant effect on beta-diversity, indicating that abiotic factors in the soil can also affect endosphere microbiome assembly (p= 0.005, 0.004, 0.006) (**Figure 6**; **Supplementary Table S1**).

#### 3.1.3 Differential abundance of specific taxa

Having observed that factors derived from both seed and soil affected the diversity of microbiota in the cannabis seedling endosphere, we sought to describe the effects of cannabis genotype, soil source, and soil sterilization on the abundance of specific bacterial taxa.

When the effect of soil sterilization was tested across all genotypes and soil sources, five taxonomic classes of bacteria showed significant differential abundance. Generally, cannabis seedlings planted in non-sterilized soil had endospheres that were enriched with *Gammaproteobacteria* [5.24x fold, W= 72], *Flavobacteriia* [47.21x fold, W=70], and *Alphaproteobacteria* [7.69x fold, W=66], as compared to sterilized soil. Conversely, endospheres of cannabis seedlings grown in sterilized soil were enriched with *Betaproteobacteria* [1.41x fold, W=65] and non-significantly with *Bacilli* (49x fold, W=5] (**Supplementary File S2**).

Each cannabis genotype was also tested individually in terms of the effect of soil sterilization and soil source on the abundance of its endosphere taxa. This analysis can help to identify genotype-specific effects on the endosphere microbiome. BC Big Bud grown in non-sterilized Okanagan soil had far more *Betaproteobacteria*, *Gammaproteobacteria*, *Flavobacteriia*, and *Sphingobacteriia* than BC Big Bud grown in sterilized Okanagan soil [12.87x, W=50, 40.39x, W=52, 47.22, W=52, 8.68x, W=50 respectively] BC Big Bud grown in non-sterilized Innotech soil had far more *Gammaproteobacteria* as compared to sterilized Innotech soil [7.73x, W=52] (**Supplementary File S2**).

The seedling endosphere of the Katani genotype grown in non-sterilized Okanagan soil had significantly more *Actinobacteria*, *Gammaproteobacteria*, *Flavobacteriia*, and *Sphingobacteriia* than Katani grown in sterile Okanagan soil [1.51x, W=42, 5.24x, W=41, 9.17x W=40, 100.48x, W=39, respectively]. Sterilized Okanagan soil from which viable microbes had been removed resulted in significantly more abundance of *Bacilli* [1.42x, W=41] Endospheres of Katani seedlings grown in non-sterilized Innotech soil were significantly enriched in *Actinobacteria*, and *Gammaproteobacteria* when compared to seedlings of the same genotype grown in sterilized Innotech soil [41.23x W=42, 8.99x W=41, respectively]. Sterilizing soil to remove environmental microbes resulted in significantly greater relative abundance of *Bacilli* [13.10x, W=41] (**Supplementary File S2**).

The seedling endosphere of the X59 genotype grown in non-sterilized Innotech soil had more *Betaproteobacteria*, *Gammaproteobacteria*, and *Flavobacteriia* than X59 grown in sterilized Innotech soil [29.23x, W=45, 10.31x, W=42, 901x W=46, respectively]. X59 seedlings grown in non-sterilized Okanagan soil were enriched in these same three taxonomies when compared to plants grown in sterilized Okanagan soil [7.78x, W=45, 11.26x, W=42, 986x, W=46]. X59 seedlings grown in sterile Okanagan soil were significantly enriched in Bacilli when compared rare taxonomies were filtered [4.39x, W=6] (**Supplementary File S2**).

#### 3.1.4 Metagenome functional prediction with PICRUSt2

The changes observed in bacterial endosphere communities when cannabis genotypes were grown with or without soil inoculum suggested that this niche may be occupied by bacteria with different functionalities, depending on their environment. Therefore, metagenome functional prediction was conducted using PICRUSt2 (Phylogenetic Investigation of Communities by Reconstruction of Unobserved States) (Douglas et al., 2013). In the first stage of this analysis, sterilized and non-sterilized soils were compared across all genotypes and soil sources using ANCOM differential abundance testing (**Table 1**) (**Supplementary File S3**). Four gene pathways were significantly different in their representation in endospheres of seedlings grown in sterilized versus non-sterilized soils. Seedlings that were germinated in non-sterilized soil were enriched in the octane oxidation (P221-PWY); a pathway commonly found and well-studied in *Pseudomonas* species (Chakrabarty et al., 1973). On the other hand, seedlings grown in sterilized soil were significantly enriched in the pathways: L-lysine biosynthesis II (PWY-2941), lactose degradation I (LACTOSECAT-PWY), and S-methyl-5-thio-α-D-ribose 1-phosphate degradation I (PWY-4361) (**Supplementary File S3**).

**Table 1:** PICRUST ANCOM differential abundance MetaCyc pathways for soil sterilization.

Gene Pathway	MetaCyc Pathway Name	Sterilized Relative Abundance	Natural Relative Abundance
P221-PWY	octane oxidation	37295	102195
PWY-2941	L-lysine biosynthesis II	158749	2468
LACTOSECAT-PWY	lactose degradation I	3648	1448
PWY-4361	S-methyl-5-thio-α-D-ribose 1-phosphate degradation I	137054	1473

All cannabis samples were pooled and compared between autoclave sterilized and natural untreated soil. Pathways shown were determined to be significantly different between treatments as determined by the W statistic.

Next, the effect of soil source and soil sterilization on the seedling endosphere was tested for each cannabis genotype separately (**Table 2**). BC Big Bud planted in sterilized Innotech soil, as compared to all other soil treatments, was significantly enriched in the methanogenesis pathway, which produces methane from H_2_ and CO_2_ (METHANOGENESIS-PWY). The same seedlings were also enriched in the reductive acetyl coenzyme A pathway I (CODH-PWY). The CODH-PWY is associated with autotrophic bacteria who can produce the energetic compound acetyl-COA by fixing CO_2_ (**Supplementary File S3**).

**Table 2:** Selected significantly differentially abundant MetaCyc pathways for genotypes in soil treatments.

	Innotech Soil	Sterile Innotech Soil	Okanagan Soil	Sterile Okanagan Soil
**BC Big Bud**		METHANOGENESIS-PWY		
		CODH-PWY		
**Katani**	3-HYDROXYPHENYLACETATE-DEGRADATION-PWY		3-HYDROXYPHENYLACETATE-DEGRADATION-PWY	
	POLYAMINSYN3-PWY		POLYAMINSYN3-PWY	
	Chitin Degradation PWY-6906		Chitin Degradation PWY-6906	
			THREOCAT-PWY	
**X59**	Peptidoglycan Biosynthesis II Pathway (PWY-5265)			Aerobic Thiazole Biosynthesis II Pathway (PWY-6891)

Each genotype was analyzed separately. Pathways shown were determined to be significantly different between treatments as determined by the W statistic. Selected pathways had highest percent abundance values of at least 400. The full table can be seen in **Supplementary File S3**.

Seedlings of the genotype Katani grown in non-sterilized soil of either source were significantly enriched in several pathways as compared to Katani seedlings grown in sterilized soil. The enriched pathways include 4-hydroxyphenylacetate degradation (3-HYDROXYPHENYLACETATE-DEGRADATION-PWY), polyamine biosynthesis (POLYAMINSYN3-PWY), and chitin derivatives degradation (PWY-6906). Only Katani seedlings grown in non-sterilized Okanagan soil were significantly enriched in the super pathway of L-threonine metabolism (THREOCAT-PWY), when compared to seedlings of this genotype grown in any other soil condition (**Table 2**) (**Supplementary File S3**).

Endospheres of X59 seedlings grown in sterilized Okanagan soil were significantly enriched in the aerobic thiazole biosynthesis II pathway (PWY-6891), which has been well-studied in *Bacillus* species. X59 seedlings grown in non-sterilized Innotech soil were significantly enriched with the peptidoglycan biosynthesis II pathway (PWY-5265) compared to other soil treatments (**Table 2**) (**Supplementary File S3**).

## 4 Discussion

The relative contributions of the seed and soil to phytobiomes have been explored in several systems, but are not yet fully understood. Juvenile maize rhizospheres obtain inoculum from both seed and soil (Johnston-Monje et al., 2016). Other research, using *Brassica napus*, showed that soil bacteria are the primary source of inoculum for seedling rhizospheres (Rochefort et al., 2021). Research has suggested that root endospheres of some plant species are distinct assemblages rather than a community subset of rhizospheres (Gottel et al., 2011). In cannabis, soil type was found to be the primary determinant for shaping cannabis microbiomes, while plant genotype plays a significant role in determining microbial community structure, particularly in the endorhiza (Winston et al., 2014). Of note, this cannabis study by Winston et al. was done with mature plants, and so the influence of seed-borne bacteria may have been overlooked.

Using 16S amplicon sequencing, we compared the effects of cannabis genotype, soil sterilization, and soil source on seedling endosphere bacteria, in terms of alpha-diversity (Shannon), beta-diversity (Bray-Curtis), and relative taxon abundance. An assumption of our analysis is that two long cycles of autoclaving was sufficient to effectively remove all viable microbes from the autoclaved soil samples. Although there are also non-bacterial contributors to the endosphere, we limited the scope of this study to bacteria for practical reasons and because of our ongoing interest in characterizing the bioactive bacteria in cannabis seedling endospheres (Dumigan and Deyholos, 2022). We found that each of the three types of treatments (cannabis genotype, soil sterilization, and soil source) had a statistically significant effect under at least some conditions, suggesting that inoculum from both seed and soil can contribute to the juvenile seedling endosphere in cannabis. We noted that variation in microbiome composition was observed even between individuals in the same treatment group, as has been previously reported for microbiome studies (Mohammed et al., 2018).

### 4.1 Seed-dependent effects on seedling endosphere

In the present study, the variety (genotype) of seed was an experimental factor comprising both the functional properties of the plant and the microorganisms it vectors from its parent. An effect of genotype on beta-diversity in rhizosphere, endorhizosphere, and phylosphere has previously been demonstrated in three types of vegetatively propagated drug-type cannabis (Comeau et al., 2020). This is consistent with our observation of a significant effect of cannabis genotype in beta-diversity (**Figure 6**, **Supplementary Table S1**). Interestingly, within a given sterilized soil treatment, PERMANOVA based on Bray-Curtis distance matrix found genotypes to be significantly different from each other (**Supplementary Table S1**), suggesting that differences in the genotypes influenced the microbial communities of their endospheres.

Our results also showed that a core microbiome of bacterial endophytes existed across samples: *Gammaproteobacteria* and *Bacilli*, the most abundant taxa, were found in all treatments (**Figures 2**, **3**). *Betaproteobacteria*, *Flavobacteriia*, and *Alphaproteobacteria* were also found across all soil treatments but typically at lower abundance. This suggests that some portion of seed-borne endophytes inhertited from the seed-bearing parent were maintained whether seedlings were germinated in the presence or absence of soil microorganisms.

### 4.2 Soil-dependent effects on seedling endosphere

We observed that the Shannon alpha-diversity of autoclave sterilized soils was significantly lower than non-sterilized soils, when all genotypes and soil sources were grouped together (**Figure 4A**). This is unsurprising as non-sterilized soil contains abundant microorganisms that can colonize seedlings and alter the endosphere microbiota. This leads to speculation about the impact of growing drug-type cannabis in soilless medium, as is common practice for licenced cultivators in Canada to limit pathogens. However, when genotypes and soil sources were considered individually, only Katani plants grown in Okanagan soil showed a significant reduction in alpha-diversity of sterilized soil compared to non-sterilized soil (**Figure 5**). This is indicative of genotype-specific factors and the presence of particular soil microganisms in the Okanagan soil source. Other genotypes grown in soil from either Okanagan or Innotech showed non-significant responses to soil sterilization, in terms of alpha-diversity. Interestingly, when all soil treatments (i.e. both source and sterilization) were considered together, and the effect of genotype was tested, drug-type genotype Katani and X59 had significantly lower alpha-diversity as compared to the two hemp genotypes: (**Figure 4B**). Further experiments would need to be conducted with additional examples of drug-type and hemp-type cannabis to see whether this difference between these two types of cannabis is generalizable.

The relative abundance of many taxa were significantly different between endospheres of seedlings grown in sterilized soil as compared to non-sterilized (**Figures 2**, **3**). Genotype had clear and significant effect on Bray-Curtis distances, however, cannabis genotypes as a whole clustered into their soil treatment groups on the PCoA (**Figure 6**). Genotypes planted in untreated Okanagan and Innotech soils were dramatically different from one another, while the genotypes grown in the sterilized soils clustered far closer together, and so it is unsurprising that they would have an effect on endosphere communities. Meanwhile, the closer clustering of cannabis genotypes grown in both sterilized Okanagan and Innotech soil is likely a result of seed-borne endophytes being the sole colonizers of niche (**Figure 6**).

There were clear differences in relative abundance of some taxa in the endosphere when cannabis seedlings were grown on sterilized versus non-sterilized soil. In the absence of viable soil inocula, cannabis seedlings were significantly enriched in *Betaproteobacteria* (1.42x) (**Supplementary File 2**). *Betaproteobacteria* are a class of gram-negative bacteria with important roles in cycling nitrogen, sulfur, and other nutrients (Kowalchuk et al., 1997; Schmalenberger and Kertesz, 2007; Falca et al., 2010). Some also have plant growth-promiting properties, and their abundance in the potato rhizoshere is cultivar dependent and plant growth-stage dependent (Falca et al., 2010). In high-starch potato cultivars, abundance of *Betaproteobacteria* in the rhizosphere increased towards the flowering stage of the plant. Perhaps cannabis-cultivars show a similar effect which leads to an enrichment of these bacteria in flowering tissue, allowing them to be vectored to seedlings, as was inferred from our results.

Seedlings grown in sterilized Okanagan soil were significantly enriched in *Bacilli*, as compared to seedlings grown in non-sterilized Okanagan soil. This enrichment was as large as 49X when all genotypes were considered as a group, and was also significant in the two hemp genotypes individually (in X59 when rare taxa (<50 frequency) were filtered), although the enrichment in BC Big Bud (1.8X) was insignifcant. The increase in *Bacilli* between sterilized and non-sterilized Innotech soil was generally not as large or significant as Okanagan soil, perhaps because of abundant *Bacilli* in the non-sterilized Innotech soil. *Bacilli* are a class of economically and agriculturally relevant bacteria, some of which promote plant growth and create a variety of antimicrobial secondary metabolites (Ongena and Jacques, 2008; Cochrane and Verderas, 2014; Hashem et al., 2019). Additionally, they form robust endospores capable of surviving harsh environental conditions for long periods of time (Nicholson, 2002; Hashem et al., 2019). Interestingly, *Bacilli* spores have been revived from 25-40 million year old amber (Raul and Monica, 1995). It is therefore unsurprising that this class of bacteria would persist in cannabis seeds (which can be dormant for several years) and be vectored across plant generations. Previous research demostrated cannabis plants vector culturable *Bacillus* species to seedlings and that these bacteria antagonize seed-borne pathogens (Dumigan and Deyholos, 2022).

*Gammaproteobacteria* showed the most dramatic differences in abundance in endospheres of cannabis seedlings grown in non-sterilized soils as compared to sterilized soils. This taxon was enriched 329x in non-sterilized soils, when all genotypes and soil sources were considered collectively. *Gammaproteobacteria* is a class of bacteria commonly associated with plants, and has even been identified as an indicator of a healthy microbiome and suppressive of wilt caused by *Fusarium* spp. in banana plants (Köberl et al., 2017). *Pseudomonas*, a genus within this class, is the source of bacteria with biocontrol activity against cannabis fungal pathogens (Balthazar et al., 2022). Interestlingly, in an previous report, *Pseusdomonas* was found to have plant growth promotion activity, but only when inoculated with a consortia of *Bacillus* and *Pseudomonas* species (Comeau et al., 2021). It is difficult to study the activity of these-non cultured *Gammaproteobacteria* (Dumigan and Deyholos, 2022), however, this previous research leads to questions about potential evolution of plant growth promoting and antifungal consortia that may be vectored on seed or obtained from soil to protect emerging cannabis seedlings.

*Flavobacteriia* and *Sphingobacteriia* were also differentially abundant and higher in endospheres of cannabis seedlings grown in non-sterilized Okanagan as compared to sterilized Okanagan soil (**Supplementary File S2**). Interestingly, this was not seen in Innotech soil, where *Flavobacteriia* were significantly enriched only in the genotype X59 when compared sterilized Innotech soil. This is likely a result of an abundance of the classes of bacteria in Okanagan soil. *Flavobacteriia* are often found in root-associated microbiomes, but the factors influencing their abundance are unclear. A recent study found that abundance of *Flavobacterium* (a genus of *Flavobacteriia*) is correlated with pectin availability in plant-associated communities (Kraut-cohen et al., 2021). Cannabis fiber is composed of 1-17% pectin (Liu et al., 2017), and perhaps soil Flavobacteriia species have evolved to associate with these plants for their pectin content. The genus *Sphingobium* from the class *Sphingobacteriia* has been well-studied for its biodegradative properties and for bioremediation (Kunihiro et al., 2013; Boss et al., 2022). They are also known antagonize pathogenic *Pseudomonas* (Takeuchi et al., 2001).

Abiotic soil characteristics could also be inferred to affect microbiome composition (**Figure 6**; **Supplementary Table S1**). This is demonstrated by the differences endosphere beta-diversity observed when seedlings were grown on sterilized soils, each from two different sources. Physical properties such as the sand, silt, and clay content may influence this, as well as the differences in organic matter, pH, and nutrient content in the Okanagan vineyard soil versus the Innotech hemp farm soil.

### 4.3 Inferred biochemical functions of the cannabis endosphere

The differences in relative abundance we observed for various bacterial taxa leads to the question of what the resulting functional characteristics of these plant-bacteria associations are. The observation that the octane oxidation (P221-PWY) pathway was enriched in non-sterilized soil as compared to sterilized soil, when all other factors were considered together, suggests seedling endospheres acquire this metabolic capacity from the soil, presumably from the well-profiled *Pseudomonas* species with the capacity to degrade this hydrocarbon (Chakrabarty et al., 1973). This could be a product of co-incidence (preferential colonization of these bacteria who happen to have this pathway), or a plant adaptation to a soil environment where octane oxidation is beneficial.

On the other hand, plants grown with only seed-inoculum were enriched in the pathways: L-lysine biosynthesis II (PWY-2941), lactose degradation I (LACTOSECAT-PWY), and S-methyl-5-thio-α-D-ribose 1-phosphate degradation I (PWY-4361). The enrichment of the lactose degradation I pathway is likely a result of the increased abundance of *Bacilli* in plants grown in sterile soil, as this pathway is well characterized and commonly found in this taxonomic class (Bissett and Anderson, 1973). PWY-4361 was 93-fold more abundant in plants grown in sterile soil compared to untreated soil (**Table 2**). This pathway functions to salvage the amino acid methionine from sulfur containing by-products of polyamine synthesis (Avila et al., 2004), or in some organisms, by-products from the synthesis of the phytohormone ethylene (Yang and Hoffman, 1984; Albers, 2009). Interruption of the this methionine salvaging pathway in Arabidopsis has a negative effect on ethylene production (Bürstenbinder et al., 2007), likely due to a build-up of the inhibitory compounds that are not being recycled (Pattyn et al., 2021). Given the critical role of ethylene in seed dormancy, and the observation that ethylene can induce germination under certain conditions (Yang and Hoffman, 1984; Wang et al., 2020), there is a possible function of these bacterial genes in regulating germination of cannabis seedlings. Perhaps the enrichment of this pathway in seed-borne cannabis endophytes has evolved to assist in seedling germination.

To get specific information about how gene pathway abundance shifts in response to soil and soil sterilization in each genotype, PiCRUST2 analysis was done individually for each genotype. BC Big Bud plants in sterilized Innotech soil were enriched with the methanogenesis pathway to produce methane from H_2_ and CO_2_ (METHANOGENESIS-PWY), indicating the presence of methanogenic archaea being vectored to seedlings via seed. Methanogens grow by converting H_2_ and CO_2_ to methane to create energy (Buan, 2018). Methanogens have been found in rice endospheres (Edwards et al., 2015), and have been suggested to contribute the methane greenhouse gas emissions (Neue, 1993). The role of these methanogens in germinating cannabis seedling endospheres needs further examination. Additionally, the reductive acetyl coenzyme A pathway I (CODH-PWY) was enriched in this environment, suggesting autotrophic bacteria that can fix CO_2_ can be vectored from BC Big Bud seeds to germinating seedlings.

Katani seedling endospheres were significantly enriched in many biochemical pathways when grown in non-sterilized soil (**Supplementary File S3**). A few of these pathways are highlighted here. The pathway for 4-hydroxyphenylacetate degradation (3-HYDROXYPHENYLACETATE-DEGRADATION-PWY) is found in a number of bacteria, including denitrifying bacteria (Seyfried et al., 1991), suggesting that denitrifying bacteria from the soil colonize plant endospheres. In Katani plants, both soil sources contributed bacteria with the gene pathways for polyamine biosynthesis and chitin degradation (**Table 2**). Polyamines are known to act as signaling molecules triggering plant defense and symbiotic plant-microbe interactions (Jiménez-Bremont et al., 2014). Chitin is a major structural compound of fungal cell walls (Beier and Bertilsson, 2013), and the presence of chitin degrading bacteria is not surprising as untreated soil would have an abundance of fungi. It is interesting that these chitin degrading bacteria are colonizing cannabis seedling endospheres. Available data suggests there are shifts in bacterial community structure towards chitinolytic bacteria in response to chitin availability (Kielak et al., 2013). It could be the germinating Katani plants are selecting for chitin- degrading bacteria from soil when grown in an environment with abundant fungi. Finally, in Katani plants grown in untreated Okanagan soil, the super pathway for L-threonine metabolism was significantly enriched compared to other soil treatments. Bacterial amino acid metabolism has recently been shown to be important to synthesize signaling molecules to associate with host plants (Moormann et al., 2022). Perhaps bacteria derived from Okanagan soil are using L-threonine to create signaling molecules to associate with Katani seedlings.

X59 plants grown in sterilized Okanagan soil were significantly enriched in the aerobic thiazole biosynthesis II pathway (PWY-6891). This pathway has been well-studied in *Bacillus* species and is likely a result of the dominance of seed-borne *Bacilli* in X59 endosphere grown in these conditions (Moormann et al., 2022). Thiazole is a major component of many naturally derived antibiotics, and so the increased abundance of this pathway may be related to disease control (Arshad et al., 2022). The role of bacterial-synthesized thiazole in germinating cannabis seedlings requires further study. X59 plants grown in sterilized Innotech soil were enriched in the peptidoglycan biosynthesis II pathway (PWY-5265). Peptidoglycan is a major feature of bacterial cell walls and crucial for biofilm formation (Garde et al., 2021). It is essential for adhesion to substrates, and this MetaCyc PWY-5265 pathway has the expected taxonomic range of the phylum *Bacillota.* The enrichment of this pathway again likely stems for the dominance of seed-borne *Bacilli* when X59 plants were grown in the absence of soil microorganisms.

Unraveling the complexities of plant-microbe interactions and their effects on plant health is a major research focus in plant science. Angiosperms have evolved to vector bioactive microorganisms across plant generations to assist in seedling establishment and plant health. This phenomenon and the benefits provided is poorly understood in cannabis. This research documents for the first time the effects of both seed and soil in shaping microbial communities in this economically relevant crop. The study demonstrates that cannabis seedlings obtain a conserved community of seed-borne endophytes across soil types and treatments, and that the abundances of these bacteria shift dramatically when exposed to soil inoculum. Additionally, soil inoculum contributes a significant amount of endosphere diversity to germinating seedlings. Metagenomic functional gene prediction suggests that these seedling endophytes have enzymatic pathways potentially useful to establishing healthy cannabis plants. Future work should further investigate these activities and subsequent benefit to cannabis plants.

## Data availability statement

The original contributions presented in the study are included in the article/**Supplementary Material**, further inquiries can be directed to the corresponding author/s.

## Author contributions

CD: Conceptualization, Formal analysis, Investigation, Methodology, Writing – original draft. MD: Funding acquisition, Project administration, Resources, Supervision, Writing – review & editing.

## Supplementary material

Supplementary Table 1 PERMANOVA Bray-Curtis Beta-diversity distance matrix significant results (bray_curtis_distance_matrix-significance.qzv). **(A)** Soil Treatment PERMANOVA, **(B)** genotype PERMANOVA.

Supplementary File S1 PERMDISP Data.xlsx.

Supplementary File S2 ANCOM TABLES.zip.

Supplementary File S3 PICRUST ANCOM TABLE.CSV.

Supplementary File S4 qiime2 metadata.xlsx.
