# Supplementary material for: Soil and seed both influence bacterial diversity in the microbiome of the Cannabis sativa seedling endosphere
Source: Front Plant Sci. 2024 Feb 21;15:1326294. doi: 10.3389/fpls.2024.1326294 (PMC10914941; doi:10.3389/fpls.2024.1326294)
Supplement: Supplementary Table 1 — PERMANOVA Bray-Curtis Beta-diversity distance matrix significant results (bray_curtis_distance_matrix-significance.qzv). (A) Soil Treatment PERMANOVA, (B) genotype PERMANOVA. [file Table_1.docx]

**Supplemental Table S1**. PERMANOVA Bray-Curtis Beta-diversity distance matrix significant results (bray_curtis_distance_matrix-significance.qzv). (A) Soil Treatment PERMANOVA, (B) Genotype PERMANOVA

(A)

| **Group 1** | **Group 2** | **Sample size** | **Permutations** | **pseudo-F** | **p-value** | **q-value** |
| --- | --- | --- | --- | --- | --- | --- |
| **BC Big Bud Innotech** | **BC Big Bud Okanagan** | 15 | 999 | 2.950 | 0.003 | 0.005 |
|  | **BC Big Bud Sterile-Innotech** | 15 | 999 | 1.701 | 0.002 | 0.004 |
|  | **BC Big Bud Sterile-Okanagan** | 15 | 999 | 2.467 | 0.001 | 0.003 |
|  | **Katani Innotech** | 15 | 999 | 1.355 | 0.138 | 0.142 |
|  | **Katani Okanagan** | 15 | 999 | 5.397 | 0.001 | 0.003 |
|  | **Katani Sterile-Innotech** | 14 | 999 | 2.278 | 0.004 | 0.005 |
|  | **Katani Sterile-Okanagan** | 15 | 999 | 3.434 | 0.001 | 0.003 |
|  | **X59 Innotech** | 15 | 999 | 1.184 | 0.186 | 0.186 |
|  | **X59 Okanagan** | 15 | 999 | 3.692 | 0.003 | 0.005 |
|  | **X59 Sterile-Innotech** | 14 | 999 | 1.969 | 0.003 | 0.005 |
|  | **X59 Sterile-Okanagan** | 15 | 999 | 3.631 | 0.004 | 0.005 |
| **BC Big Bud Okanagan** | **BC Big Bud Sterile-Innotech** | 16 | 999 | 3.336 | 0.001 | 0.003 |
|  | **BC Big Bud Sterile-Okanagan** | 16 | 999 | 2.588 | 0.014 | 0.016 |
|  | **Katani Innotech** | 16 | 999 | 4.867 | 0.001 | 0.003 |
|  | **Katani Okanagan** | 16 | 999 | 2.148 | 0.030 | 0.032 |
|  | **Katani Sterile-Innotech** | 15 | 999 | 3.760 | 0.001 | 0.003 |
|  | **Katani Sterile-Okanagan** | 16 | 999 | 5.094 | 0.001 | 0.003 |
|  | **X59 Innotech** | 16 | 999 | 4.132 | 0.001 | 0.003 |
|  | **X59 Okanagan** | 16 | 999 | 3.877 | 0.003 | 0.005 |
|  | **X59 Sterile-Innotech** | 15 | 999 | 3.897 | 0.001 | 0.003 |
|  | **X59 Sterile-Okanagan** | 16 | 999 | 5.608 | 0.002 | 0.004 |
| **BC Big Bud Sterile-Innotech** | **BC Big Bud Sterile-Okanagan** | 16 | 999 | 1.861 | 0.009 | 0.010 |
|  | **Katani Innotech** | 16 | 999 | 2.802 | 0.001 | 0.003 |
|  | **Katani Okanagan** | 16 | 999 | 5.531 | 0.001 | 0.003 |
|  | **Katani Sterile-Innotech** | 15 | 999 | 1.978 | 0.004 | 0.005 |
|  | **Katani Sterile-Okanagan** | 16 | 999 | 2.825 | 0.001 | 0.003 |
|  | **X59 Innotech** | 16 | 999 | 2.080 | 0.008 | 0.010 |
|  | **X59 Okanagan** | 16 | 999 | 4.087 | 0.002 | 0.004 |
|  | **X59 Sterile-Innotech** | 15 | 999 | 1.325 | 0.048 | 0.050 |
|  | **X59 Sterile-Okanagan** | 16 | 999 | 3.111 | 0.005 | 0.006 |
| **BC Big Bud Sterile-Okanagan** | **Katani Innotech** | 16 | 999 | 4.144 | 0.002 | 0.004 |
|  | **Katani Okanagan** | 16 | 999 | 5.751 | 0.001 | 0.003 |
|  | **Katani Sterile-Innotech** | 15 | 999 | 2.794 | 0.004 | 0.005 |
|  | **Katani Sterile-Okanagan** | 16 | 999 | 3.762 | 0.001 | 0.003 |
|  | **X59 Innotech** | 16 | 999 | 3.105 | 0.001 | 0.003 |
|  | **X59 Okanagan** | 16 | 999 | 4.441 | 0.005 | 0.006 |
|  | **X59 Sterile-Innotech** | 15 | 999 | 2.203 | 0.006 | 0.007 |
|  | **X59 Sterile-Okanagan** | 16 | 999 | 2.417 | 0.019 | 0.021 |
| **Katani Innotech** | **Katani Okanagan** | 16 | 999 | 8.611 | 0.001 | 0.003 |
|  | **Katani Sterile-Innotech** | 15 | 999 | 2.620 | 0.009 | 0.010 |
|  | **Katani Sterile-Okanagan** | 16 | 999 | 4.600 | 0.001 | 0.003 |
|  | **X59 Innotech** | 16 | 999 | 1.406 | 0.158 | 0.160 |
|  | **X59 Okanagan** | 16 | 999 | 5.822 | 0.004 | 0.005 |
|  | **X59 Sterile-Innotech** | 15 | 999 | 3.143 | 0.001 | 0.003 |
|  | **X59 Sterile-Okanagan** | 16 | 999 | 5.527 | 0.001 | 0.003 |
| **Katani Okanagan** | **Katani Sterile-Innotech** | 15 | 999 | 6.213 | 0.001 | 0.003 |
|  | **Katani Sterile-Okanagan** | 16 | 999 | 8.582 | 0.001 | 0.003 |
|  | **X59 Innotech** | 16 | 999 | 6.771 | 0.003 | 0.005 |
|  | **X59 Okanagan** | 16 | 999 | 5.444 | 0.001 | 0.003 |
|  | **X59 Sterile-Innotech** | 15 | 999 | 6.427 | 0.002 | 0.004 |
|  | **X59 Sterile-Okanagan** | 16 | 999 | 8.974 | 0.001 | 0.003 |
| **Katani Sterile-Innotech** | **Katani Sterile-Okanagan** | 15 | 999 | 3.332 | 0.004 | 0.005 |
|  | **X59 Innotech** | 15 | 999 | 2.860 | 0.003 | 0.005 |
|  | **X59 Okanagan** | 15 | 999 | 4.820 | 0.002 | 0.004 |
|  | **X59 Sterile-Innotech** | 14 | 999 | 1.765 | 0.043 | 0.046 |
|  | **X59 Sterile-Okanagan** | 15 | 999 | 4.150 | 0.003 | 0.005 |
| **Katani Sterile-Okanagan** | **X59 Innotech** | 16 | 999 | 4.040 | 0.002 | 0.004 |
|  | **X59 Okanagan** | 16 | 999 | 6.531 | 0.002 | 0.004 |
|  | **X59 Sterile-Innotech** | 15 | 999 | 3.491 | 0.003 | 0.005 |
|  | **X59 Sterile-Okanagan** | 16 | 999 | 4.775 | 0.001 | 0.003 |
| **X59 Innotech** | **X59 Okanagan** | 16 | 999 | 4.796 | 0.002 | 0.004 |
|  | **X59 Sterile-Innotech** | 15 | 999 | 2.169 | 0.015 | 0.017 |
|  | **X59 Sterile-Okanagan** | 16 | 999 | 4.673 | 0.001 | 0.003 |
| **X59 Okanagan** | **X59 Sterile-Innotech** | 15 | 999 | 4.720 | 0.006 | 0.007 |
|  | **X59 Sterile-Okanagan** | 16 | 999 | 6.331 | 0.001 | 0.003 |
| **X59 Sterile-Innotech** | **X59 Sterile-Okanagan** | 15 | 999 | 3.684 | 0.002 | 0.004 |

**(B)**

| Group 1 | Group 2 | Sample size | Permutations | pseudo-F | p-value | q-value |
| --- | --- | --- | --- | --- | --- | --- |
| BC Big Bud | Katani | 62 | 999 | 2.338869149 | 0.002 | 0.003 |
| BC Big Bud | X59 | 62 | 999 | 1.972871311 | 0.005 | 0.005 |
| Katani | X59 | 62 | 999 | 2.69872317 | 0.002 | 0.003 |
